# Formulation and *in Vitro*, *ex Vivo* and *in Vivo* Evaluation of Elastic Liposomes for Transdermal Delivery of Ketorolac Tromethamine

**DOI:** 10.3390/pharmaceutics3040954

**Published:** 2011-12-15

**Authors:** Guadalupe Nava, Elizabeth Piñón, Luis Mendoza, Néstor Mendoza, David Quintanar, Adriana Ganem

**Affiliations:** División de Estudios de Posgrado (Tecnología Farmacéutica), Facultad de Estudios Superiores Cuautitlán, Universidad Nacional Autónoma de México, Av. 1° de Mayo S/N, Cuautitlán Izcalli, Estado de México, 54740, Mexico

**Keywords:** elastic liposomes, ketorolac tromethamine, skin permeation, tape stripping, TEWL

## Abstract

The objective of the current study was to formulate ketorolac tromethamine-loaded elastic liposomes and evaluate their *in vitro* drug release and their *ex vivo* and *in vivo* transdermal delivery. Ketorolac tromethamine (KT), which is a potent analgesic, was formulated in elastic liposomes using Tween 80 as an edge activator. The elastic vesicles were prepared by film hydration after optimizing the sonication time and number of extrusions. The vesicles exhibited an entrapment efficiency of 73 ± 11%, vesicle size of 127.8 ± 3.4 nm and a zeta potential of −12 mV. *In vitro* drug release was analyzed from liposomes and an aqueous solution, using Franz diffusion cells and a cellophane dialysis membrane with molecular weight cut-off of 8000 Da. *Ex vivo* permeation of KT across pig ear skin was studied using a Franz diffusion cell, with phosphate buffer (pH 7.4) at 32 °C as receptor solution. An *in vivo* drug permeation study was conducted on healthy human volunteers using a tape-stripping technique. The *in vitro* results showed (i) a delayed release when KT was included in elastic liposomes, compared to an aqueous solution of the drug; (ii) a flux of 0.278 μg/cm^2^h and a lag time of about 10 h for *ex vivo* permeation studies, which may indicate that KT remains in the skin (with the possibility of exerting a local effect) before reaching the receptor medium; (iii) a good correlation between the total amount permeated, the penetration distance (both determined by tape stripping) and transepidermal water loss (TEWL) measured during the *in vivo* permeation studies. Elastic liposomes have the potential to transport the drug through the skin, keep their size and drug charge, and release the drug into deep skin layers. Therefore, elastic liposomes hold promise for the effective topical delivery of KT.

## 1. Introduction

Ketorolac tromethamine (KT) is a non-steroidal anti-inflammatory drug. The potency of KT is 800 times greater than commonly used aspirin [1]. Further, KT is a better analgesic agent than narcotic analgesics because of its non-addictive nature and absence of respiratory side effects (e.g., suppression of breathing) [2]. KT is commonly administered by oral, intramuscular or intravenous routes for short periods of time for the management of moderate to severe pain, including postoperative pain and visceral pain associated with cancer. The drug administered orally as 10 mg tablets shows a bioavailability of 90% with little first-pass hepatic metabolism. Nevertheless, the major drawbacks for its use include its short biological half-life (4–6 h), thereby necessitating frequent administrations to achieve the desired therapeutic effect. Frequent administrations of KT often lead to severe gastrointestinal side effects associated with cyclooxygenase inhibition; these side effects can lead to high patient incompliance [3,4]. As a consequence, the FDA issued an alert about these side effects and recommended the inclusion of medication guide [5].

KT's potent analgesic activity and low molecular weight make it a good candidate for transdermal administration. Furthermore, KT previously exhibited good cyclooxygenase-2 inhibition in a dermal fibroblast culture, showing high anti-inflammatory activity and thus increasing the potential for a useful transdermal preparation [6]. Moreover, transdermal delivery of KT would reduce the dose required to achieve a therapeutic effect and would reduce the risk of adverse events in the gastrointestinal tract.

Although transdermal administration offers diverse advantages for non-invasive drug delivery, the drug must transit the lipidic stratum corneum and cross the aqueous epidermal and dermal layers before reaching the target tissues [7]. This requirement implies that the transdermal dosage form must allow the drug to penetrate deeply into the skin, which is important considering that KT is hydrophilic in nature and its absorption through the skin is poor. Therefore, a number of different methods have been investigated in order to enhance the transdermal delivery of KT. These methods include the use of both chemical (e.g., drug permeation enhancers) and physical (e.g., iontophoresis) enhancing methods [4,6,7]. Regardless of the technology, however, the drug must be formulated so as to enable a convenient transdermal administration. In this sense, colloidal carriers represent an alternative to conventional formulations [8,9].

Cevc [10,11] proposed a carrier able to overcome the former drawbacks: Transfersomes^®^, ultradeformable lipidic vesicles, also called elastic liposomes. These vesicles are flexible with promising new uses in the transdermal drug delivery field, due to their ability to penetrate the skin in a spontaneous manner, reaching deep subcutaneous tissues while preserving their physical integrity. A combination of lipids and biocompatible membrane softeners facilitates the formation of flexible vesicles that are able to penetrate throughout the intercellular domain of the skin.

The mechanism by which elastic liposomes penetrate the skin relates to the natural transcutaneous hydration gradient. Once applied to the skin's surface, elastic liposomes dehydrate due to water evaporation, but their hydrophilicity causes the vesicles to be attracted to areas with high water content, e.g., the deep layers of the skin. The transepidermal osmotic gradient generated allows the vesicles to penetrate the intercellular spaces. This fact, as well as the ability of elastic liposomes to be deformed, results in the penetration of intact vesicles through spaces that are much smaller than their own size, carrying their load into the subcutaneous tissue [7]. Therefore, the aim of this work was to formulate elastic liposomes containing KT and to evaluate their *in vitro* drug release properties as well as their *ex vivo* and *in vivo* transdermal delivery capabilities.

## 2. Experimental Section

### 2.1. Materials

Epikuron 200 (containing 95% phosphatidylcholine) was a gift from Degussa BioActives GmbH & Co., Germany. Ketorolac tromethamine was provided by Globe Chemical México. Sephadex-G-10 and Tween 80 were purchased from Sigma Chemicals, USA. Cellophane membrane (Spectra/Por® Membrane, molecular porous MWCO: 6–8,000) was obtained from Spectrum Laboratories, Inc., USA. All other chemicals were reagent grade and used as received. Water was obtained from a Milli-Q System (Millipore^®^, USA).

### 2.2. Formation of KT-Loaded Elastic Liposomes

The elastic liposomes were prepared according to the thin-layer evaporation method. Briefly, 86 mg of Epikuron 200, 14 mg of Tween 80 (edge activator) and 4 mg of KT were placed in a round-bottom flask (these amounts were used to produce 2 mL of vesicle suspension), and the mixture was dissolved in a suitable volume of ethanol. The solvent was removed by rotary vacuum evaporation at 40 °C. Final traces of solvent were removed under vacuum overnight. The film formed was hydrated with a hydroalcoholic solution (ethanol in 7% distilled water, v/v) at room temperature with the help of a vortex for 15 min. The vesicle suspension obtained was swollen for two hours and was sonicated at room temperature for 10, 20, or 30 min at 40 kHz (Branson Ultrasonic Co., USA). The sonicated vesicles were extruded manually, either one, two, three, four or five times through 100 nm polycarbonate membrane filters (Millipore^®^, USA). The final lipid and drug concentrations were 5% w/v and 0.2% w/v, respectively.

### 2.3. Characterisation of KT Elastic Liposomes

The average diameter, size distribution (polydispersity index, PI) and zeta potential of liposomes were determined by Photon Correlation Spectroscopy (PCS) using a Zetasizer (Zetasizer 3000, Malvern, UK, using the software from Malvern Instruments Dispersion Technology and Light Scattering Systems, version 1.32). Measurements were carried out in triplicate by diluting the samples with distilled water.

Surface morphology of the elastic liposomes was examined using transmission electron microscopy (TEM). To prepare samples, a drop of the vesicle dispersion was applied onto a formvar-coated copper grid and left in contact for 2 min to form a thin film. A drop of phosphotungstic acid (1%) was added, wiping off the excess with filter paper. The grid was allowed to dry and samples were examined and images were taken using a transmission electron microscope (JEOL, JEM-100CX II).

Liposome elasticity was determined by measuring the size of the vesicles before and after manual extrusion through a polycarbonate microporous membrane with a pore size of 50 nm (Isopore, Millipore^®^). The experiment was carried out in triplicate. Conventional, non-elastic liposomes (prepared by the method described in point 2.2, excluding the surfactant) were used as a control.

To evaluate the long-term stability of KT elastic liposomes, liposomes without preservatives were stored at 4 °C for one year. At different time intervals, dispersions were subjected to macroscopic observation to find any evident sedimentation or aggregation. At the same time, particle size and polydispersity index were measured by PCS using a Zetasizer 3000 (Malvern, UK). Simultaneously, the ability of elastic liposomes to retain the drug was assessed by keeping the elastic liposome preparation at 4 °C. Samples were analysed for drug entrapment at different periods of time (1–11 weeks) by High Performance Thin Layer Chromatography (HPTLC) as described in Section 2.4.

### 2.4. HPTLC Analysis

Samples were analyzed for KT content by HPTLC. KT content was determined by spotting samples on TLC plates (Alugram® Sil G/UV_254_, Macherey-Nagel, Germany) using the Automatic CAMAG TLC Sampler III (version 2.12, Switzerland). Standard solutions of increasing concentrations were applied for mass calibration. Separation was carried out in a developing chamber (CAMAG, Switzerland) saturated at room temperature with ethyl acetate:chloroform:acetic acid (8:3:0.1). After drying the plates, the plates were scanned with a CAMAG TLC Scanner 3 at 323 nm. According to the test required, the matrices of the samples had to be different (*i.e.*, for stability studies the solvent was ethanol; for entrapment efficiency, water; for *ex vivo* studies, pH 7.4 phosphate buffer solution; for *in vivo* studies, methanol). In each case, the method was validated using the corresponding solvent.

### 2.5. Drug Entrapment Efficiency of Elastic Liposomes

To determine the entrapment efficiency, free drug (not incorporated into the vesicles) was separated from the liposomes by gel permeation chromatography with a Sephadex G-10 column using the mini-column centrifugation method described elsewhere (12–14). Briefly, Sephadex G-10 was swollen in distilled water at ambient temperature with occasional agitation, for at least six hours. The gel obtained was then stored at 4 °C. The mini-column was prepared by placing a filter paper pad in the base of a 3 mL syringe and filling it with the gel. Any water excess was removed by centrifugation at 1500 rpm for three minutes. Next, 200 μL of the vesicle suspension was applied to the mini-column and centrifuged (1500 rpm for 3 min). Next, 400 μL of water was added and the centrifugation was repeated to elute the vesicles. It is important to mention that at this stage, free drug remained in the column and the eluate did not contain KT (this was previously proven by applying a saturated solution of KT instead of the vesicle suspension and repeating the same procedure; under these conditions, no drug was eluted). Once free KT was separated from the elastic liposomes, the percentage of KT entrapped in the liposomes was determined by disrupting the vesicles with ethanol and using HPTLC (as described in Section 2.4) to quantify the amount of KT released. Entrapment efficiency (EE%) was calculated according to Equation (1).

(1) EE%=(Entrapped drug/Total drug)×100

### 2.6. Evaluation of *in Vitro* KT Release from Elastic Liposomes

*In vitro* drug release was evaluated using Franz diffusion cells. A cellophane dialysis membrane with molecular weight cut-off of 8000 Da (Spectra/Por®) was hydrated with the receptor medium (pH 5.5 phthalate buffer) for 12 h before being fastened between the donor and receptor compartments. The donor medium consisted of either 1.5 mL of the elastic liposome suspension or a 2 mg/mL KT aqueous solution (this later was used only for comparison purposes). The receptor compartment was filled with 20 mL of pH 5.5 phthalate buffer and stirred with a magnetic bar. The available diffusion area was 5 cm^2^. The temperature of the assay was controlled at 32 °C to mimic human skin. Four-milliliter aliquots were withdrawn at fixed time intervals and immediately replaced with an equal volume of fresh buffer. All samples were analyzed for KT content by spectrophotometry at 323 nm. No interference was found for any of the other components when a control test was done with empty liposome. The experiment was done in triplicate.

### 2.7. *Ex Vivo* Skin Permeation of KT

Experiments were performed under non-occluding conditions. Briefly, dermatomed (500 μm thickness) pig's ear skin (provided by a local slaughterhouse) was mounted between the donor and receptor compartments of vertical Franz-type diffusion cells with an effective permeation area of 1.5 cm^2^. The receptor solution consisted of 2 mL of pH 7.4 phosphate buffer, constantly stirred with a magnetic bar. Skin surface temperature was maintained at 32 °C. Then, 200 μL of the elastic liposome suspension containing KT was added to the donor compartment. Five hundred-microliter aliquots of the receptor fluid were withdrawn periodically over 24 h, replacing them with an equivalent volume of fresh solution, and analyzed for drug content by HPTLC. At the end of the experiment and after the skin was cleaned 5 times with a cotton cloth soaked in methanol, the skin was finely divided and immersed for 24 h in 6 mL of methanol under constant stirring at room temperature to extract the drug. Extraction suspensions were centrifuged, and the supernatants were analyzed by HPTLC to determine drug deposition in the skin. Permeation experiments with a KT aqueous solution (2 mg/mL in a 7% v/v ethanolic solution) were carried out in the same manner.

### 2.8. *In Vivo* Skin Permeation of KT

*In vivo* permeation studies were carried out by the tape-stripping technique. The study is notable for its short duration, noninvasive nature and realistic conditions. Six healthy volunteers (2 females and 4 males, skin types 3 and 4 [Fitzpatrick classification], aged 22–32 years) with no history of dermatological disease participated in this study. The nature and purpose of the study were explained to the volunteers, and informed written consent was obtained from each participant. The subjects were required to maintain the ventral forearm free from any application of cosmetic or pharmaceutical topical formulation for at least 12 h before the study and during the study.

A perfusion cell placed on the inner forearm of the volunteers. A volume of 1.5 mL of the elastic liposome suspension was added to cover the entire area (9.62 cm^2^) and the arm was wrapped with a polyester film, in order to fix the cell to the arm. The polyester film was removed from the exposed area of the cell with the intention to have a non-occlusive system. After 60 min of contact, the cell was removed, and the skin surface was wiped clean with a water-soaked cotton ball and dried gently with a gauze pad. Tape strips (Scotch Book tape No. 854 3M, USA) of equal size were cut and weighed. Each pre-weighed tape strip was placed on the treated surface area with homogeneous pressure and removed at once using forceps. Up to 16 strips were taken from each treated site such that the stratum corneum was never completely removed. The direction of strip removal was alternated from left to right. Tape strips were then weighed, and the amount of KT from each tape was extracted with methanol and quantified by HPTLC (as described in Section 2.4). The first tape was discarded, the following five tapes were extracted individually, and the remaining strips were extracted in groups of two in order to reach the detection threshold of the method.

### 2.9. Data Analysis and Statistics

Values were expressed as mean ± SD. The results were analysed statistically using analysis of variance (ANOVA). Significance was determined at P < 0.05. Duncan's test was used to determine those average values that differed significantly from the set of averages when a significant *F*-value was found.

## 3. Results and Discussion

### 3.1. Preparation and Characterisation of KT-Containing Elastic Liposomes

**Figure 1.**
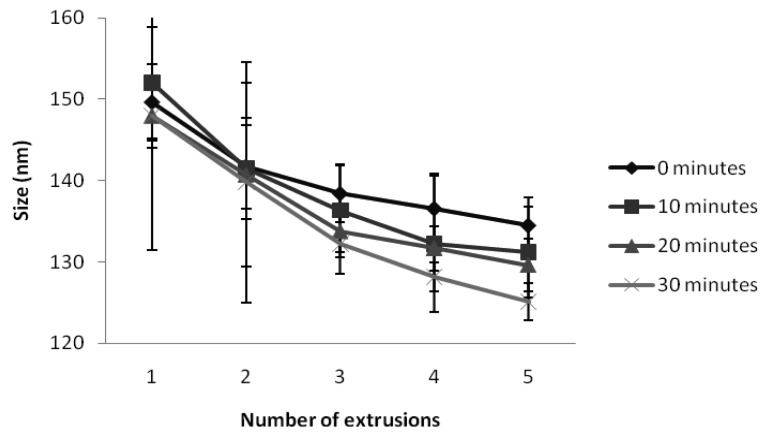
Influence of sonication time and number of extrusions on vesicle size.

A wide variety of conditions have been proposed to prepare elastic liposomes, including various sonication times (from no sonication to 60 min), number of filtrations (up to 30 times), and pore sizes of the filtering membrane. The effectiveness of pressure homogenisation to form elastic liposomes with desired size has also been reported [7,13–15]. In this study, the method to prepare KT-containing elastic liposomes was optimized by evaluating the influence of sonication time and number of filtrations. Three sonication times were assayed (10, 20, and 30 min), and vesicles were extruded up to 5 times through polycarbonate membranes with a pore size of 100 nm. Figure 1 shows that, as expected, vesicle size decreased with the number of filtrations. Significant differences (P < 0.05) were found when a bifactorial analysis of variance was carried out. The Duncan's multiple range test showed no differences when three to five extrusions were performed. Regarding sonication time, the vesicle's size was reduced as the sonication time increased, but no statistical differences were found. Therefore, optimal manufacturing conditions for KT-loaded elastic liposomes were sonication for 10 min followed by three extrusions through a polycarbonate membrane. Elastic liposomes are basically composed of a mixture of phospholipid and surfactant in an optimal proportion to give elasticity to the membrane. Different proportions of these two components have been investigated, but most studies concluded that the best ratio is 85:15 (phopholipid:surfactant). It has been reported that entrapment efficiency decreases as the surfactant proportion increases, and that elasticity diminishes with lower surfactant proportions [13,16]. For this reason, the phospholipid:surfactant ratio was maintained at 86:14 in this study.

**Figure 2.**
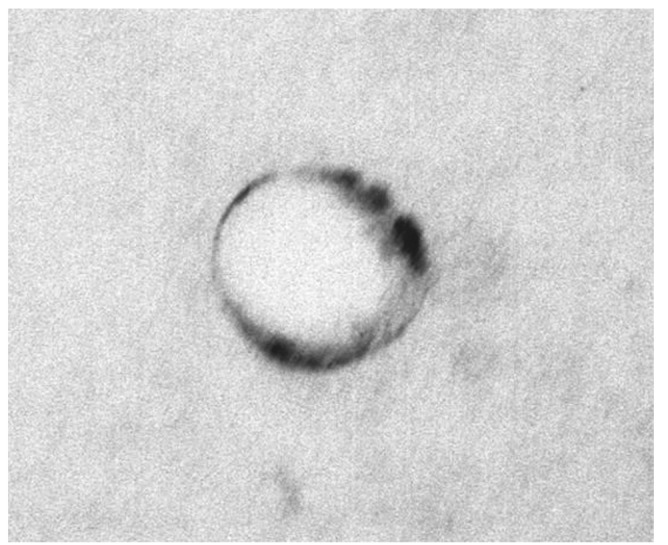
Transmission electron microscope (TEM) image of Ketorolac tromethamine (KT)-loaded elastic liposome (X 50,000).

Particle size analysis indicated that the average size of the elastic vesicles prepared was 127.8 ± 3.4 nm with a unimodal size distribution and a very low polydispersity index of less than 0.1, indicating a narrow size distribution. Rigid liposomes had a mean size of 152.7 ± 1.7 nm. The results demonstrated that incorporation of Tween 80 into elastic liposomes led to a slight decrease in particle size. Previous reports have analysed the influence of vesicle size on the ability of vesicles to penetrate into and through the skin. Vesicles up to 300 nm are able to release their cargo in the deep layers of the skin [17]. Therefore, liposomes of smaller size are considered potentially useful for the delivery of drugs through the skin. The morphology of elastic liposomes was examined by TEM. As shown in Figure 2, the liposomes appeared as unilamellar, spherical shaped vesicles.

A zeta potential of −12 mV obtained in the current study is consistent with previous reports where Tween 80 was used as edge activator and implied a good physical stability [14]. High zeta potential values, either positive or negative, are expected to render a more stable system due to a strong electrostatic repulsion. Although elastic liposomes exhibited a moderate zeta potential in this study, they were shown to be stable for one year. This stability could be attributed to the steric stabilization provided by Tween 80. It has been reported that if alkyl chains are present on the vesicle surface, the hydrocarbon chain of surfactant could penetrate into the phospholipid bilayer, exposing the polyethylene oxide groups on the surface of the vesicles, thereby producing a steric stabilization, which could decrease vesicle fusion [18].

The results presented evidenced the ability of these vesicles to be deformed due to the presence of Tween 80, which enabled the liposomes to change their shape under stress without rupture. This effect could be attributed to the propensity of Tween to form highly curved structures (e.g., micelles), thus diminishing the energy required for globule deformation. The size of elastic liposomes (either KT-containing liposomes or encapsulated + free KT liposomes) containing Tween 80 changed slightly after passing them through a membrane with a pore size of 50 nm. No differences were found between these two kinds of vesicles, since both of them showed the same capability to pass through the membrane. Table 1 shows the average sizes obtained before and after passing the vesicles, through this membrane. Then, due to their flexibility, elastic liposomes can squeeze through the intercellular regions of the stratum corneum, being able to penetrate pores that are five-fold smaller than their own diameter [19,20]. In the case of conventional liposomes, they were not able to pass through the membrane (pore size of 50 nm) when manual pressure was applied. These results agreed with reports published elsewhere [16].

**Figure 3.**
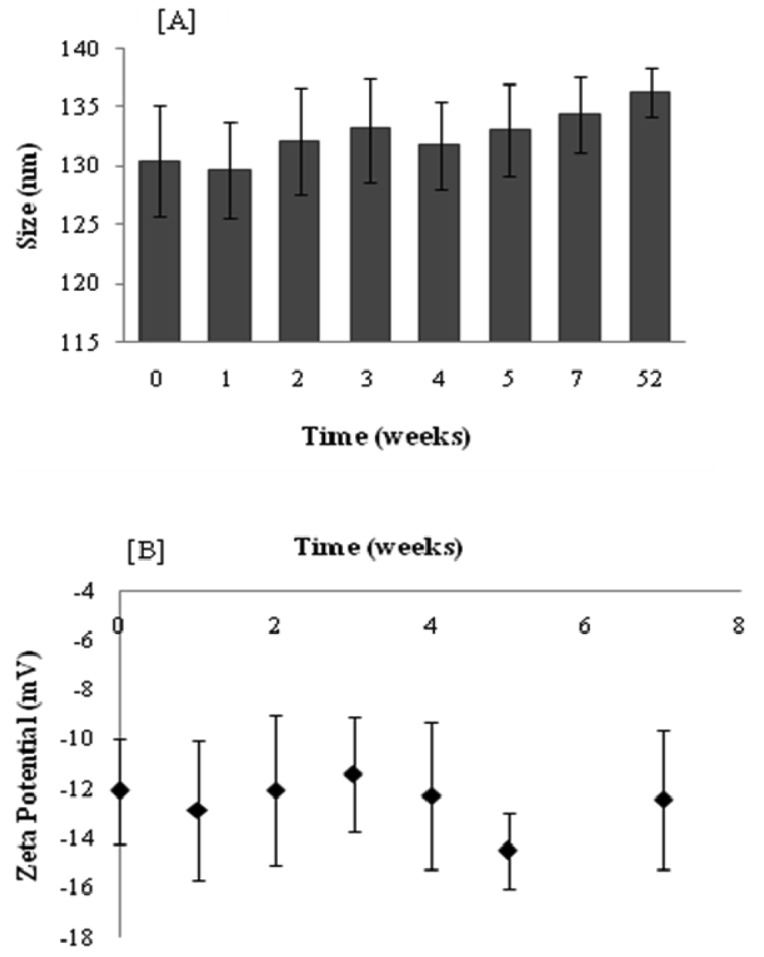
Size (**A**) and Z potential (**B**) of elastic liposomes as a function of time for determination of physical stability.

**Figure 4.**
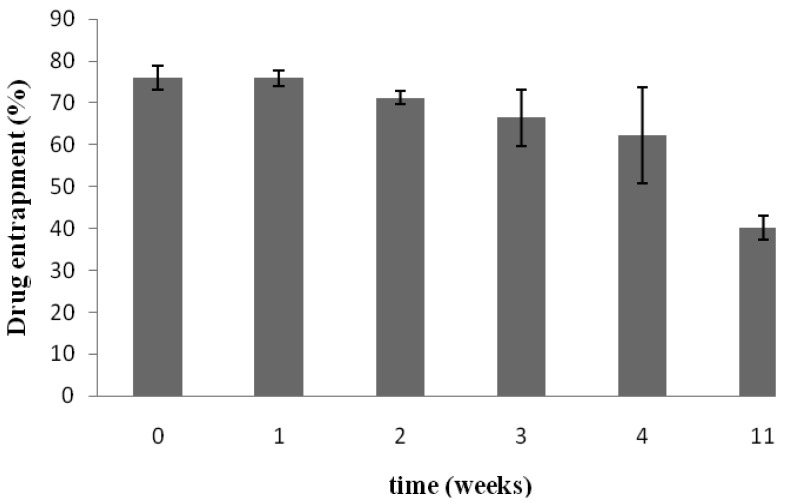
KT leakage from the elastic liposome formulation during storage (n = 4).

Figure 3A shows that under the storage conditions tested, elastic liposomes were stable without significant changes in vesicular size after one year of storage at 4 °C (size increase ∼4.5%). Figure 3B presents the zeta potential values that were monitored over a period of seven weeks. The zeta potential of elastic liposomes stored at 4 °C remained constant at around −12 mV. However, even if size and zeta potential did not change, the drug content fell ∼52% after 11 weeks with regard to the initial content (Figure 4). There were no significant differences between the first 3 weeks (Duncan's Test). In general, aqueous liposomal dispersions may suffer a series of stability problems such as aggregation and leakage of the encapsulated drugs. In this sense, freeze-drying may offer an alternative for storing liposome suspension, by preserving them in a more stable dry state [21].

### 3.2. Entrapment Efficiency

Entrapment efficiency is the fraction of KT incorporated into the elastic liposomes compared to the total amount of KT. The results showed that the maximum amount of KT incorporated into the vesicles was approximately 18.6 μg/mg of phospholipid (corresponding to an entrapment efficiency of 73.13 ± 11.13%). This entrapment efficiency is comparable to those obtained for other hydrophilic drugs, such as ketotifen fumarate formulated in elastic vesicles, using Tween 80 as an edge activator [22].

### 3.3. *In Vitro* Drug Release

**Figure 5.**
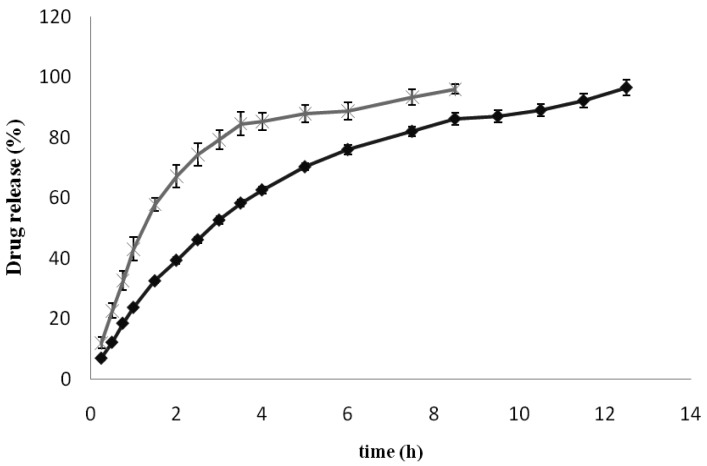
*In vitro* release profiles of KT from elastic liposomes (◆) and an aqueous solution (×) (means ± SD, n = 3).

The role of the carrier (in this case elastic liposomes) is of particular importance, since it must efficiently release the drug and facilitate drug transport across the skin. The release of KT encapsulated in the elastic liposomes (free KT was previously separated as indicated in Section 2.5) was evaluated using vertical Franz diffusion cells with a synthetic membrane between the donor and receptor compartments. The use of Franz cells provides an accurate and reliable method for evaluating active compound release from topical formulations [23]. Figure 5 shows the *in vitro* release profile of KT from elastic liposomes and from an aqueous solution using a pH 5.5 phthalate buffer as the release medium. As can be seen, elastic liposomes delayed KT release, making them slower than the aqueous solution.

The diffusion of an entrapped molecule from a disperse system is governed by the transfer of the molecule from the delivery system to the external aqueous phase and by the diffusion of the molecule through the dialysis membrane into the release medium of receptor compartment. Only the molecules present in the external aqueous phase are able to permeate through the membrane [24,25]. As shown in Figure 5, the control formulation (KT solution) exhibited a rapid drug release behavior with 80% of KT released after 3 h. In contrast, KT-loaded elastic liposomes exhibited a more controlled drug release pattern over a period of 13 h.

### 3.4. *Ex Vivo* Skin Permeation of KT

**Figure 6.**
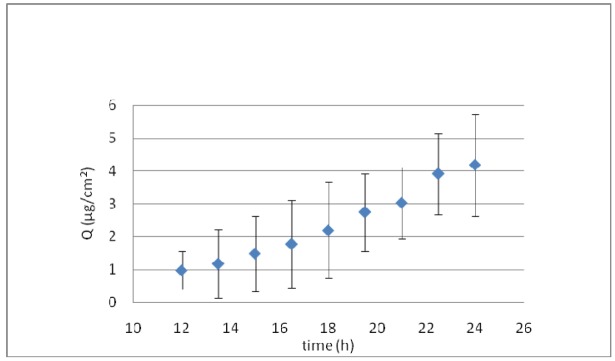
Cumulative amount of KT (Q) permeated through the skin *ex vivo* from elastic liposomes (means ± SD, n = 5).

Figure 6 shows the *ex vivo* skin permeation of KT formulated in elastic liposomes. A long lag time of about 10 h was obtained, suggesting that KT-loaded elastic liposomes could exert a local effect, as indicated by 12 h drug retention in the skin before reaching the receptor solution. After this period, a constant flux of 0.278 μg/cm^2^h was obtained. However, at the end of permeation, the residual amount of KT in the skin was of 12.61 ± 2.19 μg, and the total amount permeated was 10 μg. Therefore, after 24 h of permeation, about 269 μg remained in the donor solution (taking into account that 292 μg were placed in the donor solution at the beginning of the permeation) and only ∼7.74% was able to permeate the skin.

The findings of the current study are in agreement with those reported by Elsayed and co-workers, who evaluated the *in vitro* permeation of ketotifen formulated in deformable liposomes through rabbit pinna skin using Tween 80 as the edge activator. The authors found that drug encapsulation of hydrophilic drugs can result in a slow release on the skin surface where phospholipids can form a lipid barrier [19]. In our case, permeation was performed only with encapsulated KT (non-encapsulated KT was previously separated, as explained in Section 2.5), and during permeation a lipid film was formed on the skin once the formulation was dehydrated, hindering drug release. This phenomenon could explain the low percentage permeated into and through the skin. These results agreed with other authors, such as Bahia and co-workers, who found that elastic vesicles formed by phosphatidylcholine and sodium cholate promote calcein permeation in essentially its non-encapsulated form [26]. Furthermore, Cevc *et al.* [27] determined that drying of the carrier formulation containing ketoprofen on the skin surface increases drug-carrier association, resulting in a decrease of its permeation through the skin. In the present study, permeation experiments with a hydroalcoholic solution of KT revealed a negligible amount of the drug in the receptor solution (below the quantification limit). However; the residual amount of KT in the skin at the end of permeation was higher for the drug solution (70.15 ± 24.25 μg) than for KT encapsulated in elastic liposomes (12.61 ± 2.19 μg). These results are in accordance with those found by Nagarsenker *et al.* [8] who reported that inclusion of KT in conventional liposomes significantly reduced the transport of the drug through *in vitro* pig skin. One possible explanation for the low accumulation of KT in the skin upon application of elastic liposomes, is related to the experimental conditions, since vesicles remain under non-occlusive conditions for an extended period of time at 32 °C, promoting their fusion and excessive dehydration, and forming a film on the surface of the skin, creating a barrier that prevents further penetration of KT.

### 3.5. *In Vivo* Skin Permeation of KT

**Figure 7.**
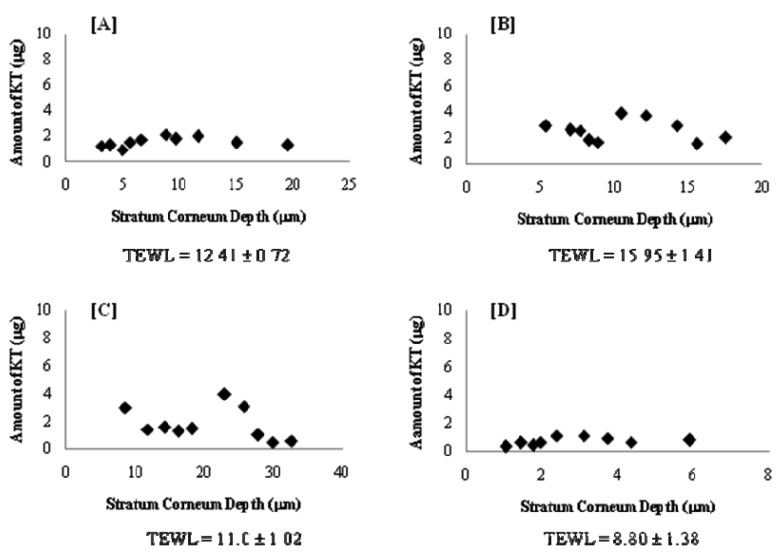

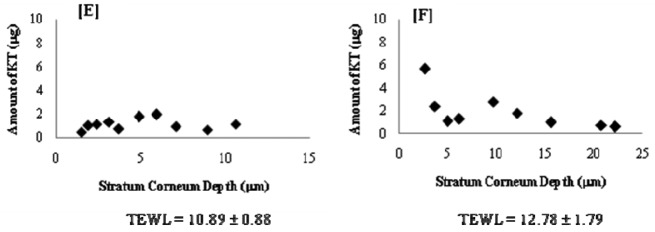
Amount of KT *vs.* stratum corneum depth after 1 hour of treatment for volunteers (**A**), (**B**), (**C**), (**D**), (**E**) and (**F**). TEWL (Transepidermal water loss, g/m^2^h) measurements were recorded before treatment.

Most of the *in vivo* studies with elastic vesicles have been conducted in animals (e.g., mice, rats, rabbits). For studies in human beings, the tape-stripping technique has gained wide acceptability as a facile and minimally invasive method to evaluate skin drug permeation. Several reports are available in the literature [28,29] on this method. In this work, *in vivo* permeation of KT was evaluated using a well-established tape-stripping technique on six healthy human volunteers. As differences in stratum corneum cohesiveness results in different amounts of stratum corneum removed from each volunteer and therefore different stratum corneum depths, the quantity of KT in the stratum corneum as a function of depth in the skin is presented individually in Figure 7. Although the drug penetration profile showed a high inter-individual variability, this could be attributed to the individual skin type and to the barrier properties of each person. For this reason, Transepidermal Water Loss (TEWL) was recorded for all volunteers prior to treatment and as shown in Figure 7, TEWL values varied from 8.8 to 15.95 g/hm^2^. Therefore, it was not surprising to find inter-individual variability, taking into account the penetration mechanism of elastic vesicles and its relationship to hydration gradient.

**Figure 8.**
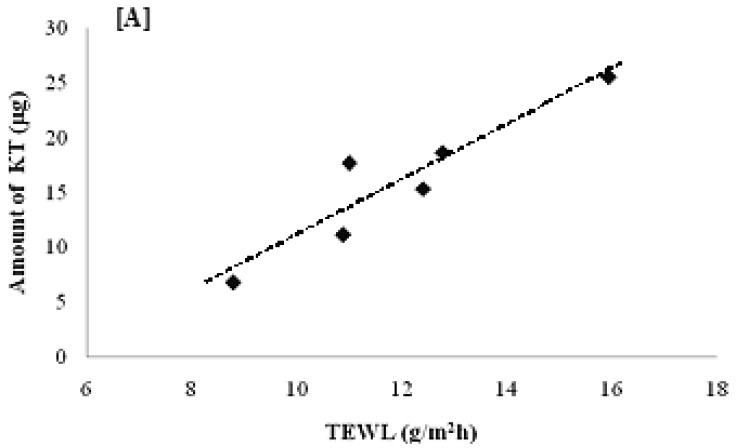

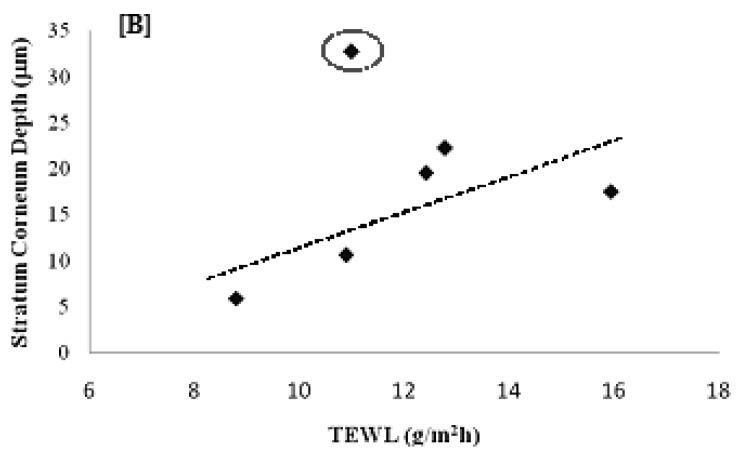
Correlation between volunteer transepidermal water loss (TEWL) value and (**A**) total amount of KT permeated; (**B**) penetration distance. The lines are from simple linear regression. In (**B**), the point marked in red was not taken into account for r^2^ calculation.

According to the results shown in Figure 7, the total amount of KT permeated for the six volunteers was 7–26 μg with a penetration distance of 6–33 μm. Interestingly, the total amount permeated (Figure 8A) and the penetration distance (Figure 8B) *vs.* individual TEWL values revealed a good correlation. Despite the obvious variability associated with *in vivo* permeation experiments, linear regression of the data resulted in a good r^2^ value (0.8779) for the amount permeated and an average r^2^ (0.5266) for the penetration distance into the stratum corneum. Although the dependence of transdermal absorption of drug upon skin hydration has already been demonstrated (a linear relationship between transdermal penetration and TEWL at the site of absorption was demonstrated [30]), the present work confirmed that the penetration mechanism of elastic liposomes correlated to the transcutaneous hydration gradient.

The differences found between *ex vivo* and *in vivo* studies were related to the conditions of the test, the viability of the tissue and the inter-species characteristics. After 1 h of contact, a total amount of 15.93 ± 6.42 μg of KT penetrated into the stratum corneum *in vivo*. Although the conditions of the *ex vivo* and *in vivo* tests made comparison difficult with this single time-point, a punctual flux of 1.65 ± 0.66 μg/cm^2^h was estimated. This value was six fold greater than the flux through excised pig skin (0.278 ± 0.1 μg/cm^2^h) after 12 h of contact. *Ex vivo*, a long contact time resulted in dehydration of the formulation with the formation of a film, hindering elastic liposome penetration and drug release. Similar results were reported by Lodén *et al.* [31], who studied the *in vitro* and *in vivo* permeation of ketoprofen formulated in a gel.

## 4. Conclusions

KT-loaded elastic liposomes were successfully prepared with an excellent loading efficiency of about 73%. The KT-loaded elastic liposomes exhibited adequate size, stability and flexibility characteristics. Furthermore, elastic liposomes extended the KT release time, achieving sustained release for almost 12 h. *In vivo* experiments demonstrated an interesting correlation between the permeation capability of elastic liposomes and the TEWL of volunteers: high TEWL values resulted in better penetration (*i.e.*, greater amount of drug and deeper penetration distance). These results confirm that penetration of elastic liposomes effectively correlate to the natural transcutaneous hydration gradient. Thus, in an ideal situation, elastic liposomes should be able to transport the drug through the skin, keep their size and drug charge, and release the drug into deep skin layers. Elastic liposomes are a potentially suitable carrier for the transdermal delivery of KT. In-depth studies should be performed to confirm their therapeutic efficacy.

## Figures and Tables

**Table 1.** Average size (nm) of elastic and rigid liposomes before and after manual extrusion through a polycarbonate membrane with a 50 nm pore size (n = 9).

**Formulation**	**Before**	**After**
Elastic liposomes without KT	130.12 ± 2.917	110.07 ± 2.903
Elastic liposomes with KT (encapsulated and free)	127.88 ± 3.435	109.57 ± 2.641
Elastic liposomes with KT (only encapsulated)	129.6 ± 4.084	100.13 ± 2.480
Rigid liposomes without KT	152.7 ± 1.749	----
